# Tracking One’s Own Path – a Methodological Leitmotif of Cultural Psychology

**DOI:** 10.1007/s12124-019-09479-2

**Published:** 2019-03-05

**Authors:** Thomas Slunecko

**Affiliations:** Department of Basic Psychological Research and Research Methods, Faculty of Psychology, University of Vienna, Liebiggasse 5, A-1010 Vienna, Austria

**Keywords:** Cultural psychology, Reflective methodology, Situated knowledge, Discourse analysis

## Abstract

Theoretically as well as alongside empirical research, this paper outlines a reflective research stance that has been thriving among cultural psychologists in Vienna since the turn of the millennium. The focus is on a specific methodological facet of this stance tentatively termed ‘tracking one’s own path’ – a metaphor for taking the starting point of research in one’s own life world, for a re-embedding of reasoning into the socio-cultural context out of which we reason, and thus for situated knowledge. The text brings forward epistemological considerations from deconstruction and phenomenology to support the methodological argument and unfolds implications for teaching and research. Three connected examples are provided to document how such research can be conducted within a critical discourse analytical frame. Upon this basis, the final section discusses the potential of the approach for cultural psychology, in particular in regard to conceptualizing an encompassing meta-theoretical frame and a coherent research programmatic.

*I can only bring to you what I’ve been going through*

Since the turn of the millennium, Vienna has seen the unfolding of an active cultural-psychological research space both at the University of Vienna and, later, at the Sigmund Freud Private University in Vienna (cf. Slunecko et al. 2017), a characteristic leitmotiv of which is elaborated in the present text. This motif is responsible for some of the epistemological and methodological – and sometimes even therapeutical – flavour of cultural psychology *à la Viennoise*. The wording that I coined for it in my habilitation treatise – it is actually the sub-title of this treatise (Slunecko 2008) – was‚ observations on one’s own path’ [*Beobachtungen auf der eigenen Spur*]. In order to avoid the somewhat detached connotation of the noun ‘observation’, I prefer for the time being to rather speak of ‘tracking one’s own path’.

Partly, this motif may derive from my psychoanalytic socialization^1^ out of which came the experience and habitus that recapitulating one’s own life path does not lead to self-encapsulation or narcissistic self-reflection but exactly beyond those. And that, in particular, someone who has discovered herself in her psychogenetic trajectory will also have more to say in regard to scientific matters.

## Elective Affinities to Deconstruction and Phenomenology

Variations of this stance are to be found in many facets of deconstructivist thinking. Deconstruction means, after all, to cancel hitherto exisiting validities. Yet, the aim of this move is not to annihilate or devaluate past experience, knowledge, and creations, but rather to re-open them, through the very cancellation of their tightly screwed validity, for new possibilities, practices, and interpretations. We look back on the totality of our past, in order „to make new use of all that was or to live, for the first time, what has not been lived in it” (Agamben 2015, p. 40, my translation^2^). Such living, process-oriented self-referencing is precisely the aim and ambition of my plea for tracking one’s own path. In the end, the retrospection and recapitulation is all about interrogating and grasping the present, a present that otherwise escapes us and eludes our understanding. As in a genuinely understood psychoanalysis, the historical ambition is always conjoined with a therapeutical ambition – a linking of (re-)search and healing, as Freud (1927) has put it – and the arrow of intention is always pointing to the future.

To borrow a metaphor from the German neophenomenologist Hermann Schmitz, we are first and foremost locked in a jungle of cultural and individual conditionings that cannot “by virtue of a mere decision on our side be transformed into an open, unprejudiced field” (Schmitz 2007, p. 11; my translation^3^). Rather, the jungle has to be arduously cleared, the hitherto valid has to be sent through the mills of deconstruction. This process is necessary, because we cannot access its truth directly, but have to go through a critique that destroys its primary affirmative security. This fundamental criticism does not erase what has been in place so far, but rather transforms and makes it accessible to the present. Stuart Hall concisely formulates this principle when he asserts of existing bodies of knowledge that “the line which cancels them, paradoxically, permits them to go on being read” (Hall 1996, p. 1).

In his *Essays after Heidegger*, Peter Sloterdijk formulates a similar idea: Philosophy, he maintains, is no longer possible without self-disclosure, and can only happen as a kind of a “conscious residence within motion” (Sloterdijk 2001, p.31, my translation^4^). And whoever wants to think from within motion “has to demonstrate what it means to volunteer as a case example” (Sloterdijk 2001, p. 31, my translation^5^). Already in one of his most seminal early books, *Weltfremdheit,* Sloterdijk had pointed to this argument: „Whoever gives a sufficiently radical account of the coming-into-being of a single individuum, reaches the point where the history of the case has to be reported as the history of being” (Sloterdijk 1993, p. 242, my translation^6^).

For Jean-Paul Sarte it was clear, too, that the individual carries a truth that extends beyond the individual. In his monumental treatise on Gustave Flaubert (Sartre 1981), for example, he reconstructs the epoch that made Flaubert’s biography possible, retotalizing from an individual drama the forces of the epoch that are embedded in this drama, ‘waiting’, so to speak, to be etched into relief. In a similar vein, the poet Cioran (1979, p. 87, my translation^7^) holds that „the only way for us to set out for the universal is to exclusively concern ourselves with what matters to us”.

The world may be all that is the case, as Wittgenstein has said; but everyone finds himself initially as – and in – his own case. The explication and development of one’s own case is everyone’s first and foremost task. By a reflected repeating of one’s own case one can raise it, gradually, to the sphere of the general (Slunecko 2008, p. 14/15). The explication of what is initially entrapped in the ‚subjective’ and individual gives rise to objectivity (in the sense of traceability and comprehensibility by others) and generalizability. By recapitulating my own case history, by reflecting upon what at first glance had seemed accidental or my own fault, I develop exactly the contribution to the general that arises from and is authenticated by my own existential position – a position that only I can hold as a result of the accumulation of *my* life experiences. In order to harvest such experience for the realm of science, a social scientist has to understand herself and what has happened to herself as “part of the weave” (a notion that I owe to Chimirri et al. 2015), not as someone – or some*thing* – apart and aloof from the world.

Instead of ‘just observing’ from a neutral third-person perspective, one has to be willing to display or confront one’s own history as one of sometimes unplanned twists and turns. But only by virtue of such confessions can we explicate the particular fold of being in which we find ourselves. ‘Plica’ is the Latin word for fold or pleat; ‘to explicate’ is, thus, best rendered as ‚to unfold‘. To explicate one’s own case history means to unfold it, and it is by such unfolding that that we arrive at a deconstructivist notion of freedom (Schubert 2018) – a freedom that does not coincide with the ability of strong subjects to choose the best alternative available to them (this is the kind of freedom the powers that be want us to believe in), but one that comes out of a reflexive critique of one’s own subjectivization and of the sociogenetic principles of knowledge production that go along with it (Bourdieu and Wacquant 1992).^8^

## Methodological and Epistemological Considerations

In contemporary human and social sciences, all this far from self-evident. Academic socialization in these fields, especially in psychology, strongly discourages such an attitude. The idea that an exposing of one’s own path should enhance one’s scientific credibility runs counter to the classic occidental conception of truth: a ‘high-altitude truth’, whose altitude precisely results from an abstaining from the knower and from the situation of the knower in order to be valid everywhere and anytime. To this kind of truth nothing personal can be contributed. Anything personal would result in its contamination. One can only take notice of such truth, accept it as a given. Such truth does not need to be *unfolded* by human agents, it can only be *found* by them.^9^

### De Nobis Ipsis *non* Silemus

In the early seventeenth century, Francis Bacon tried to specify the way to such truth in his *Novum Organum* (Bacon 1620). The ‘*Novum’* in the title indicates that something is to be renewed. What shall be renewed is Aristotle’s *Organon*, the first treatise of methods in the occidental history of ideas. In its essence, Bacon’s renewal conists of a systematization of the way to gain knowledge, a systematization through experiment. For my concern here it is important that in Bacon’s account the experimenter is imagined as a kind of univolved, if not invisible, bystander. The key sentence that Bacon makes the experimentalists take note of reads ‚*de nobis ipsis silemus*‘ – ‚we are silent concerning ourselves‘ (Kant enthusiastically borrows this motto for to the second edition of his *Critique of Pure Reason)*. The contrast with what I am proposing in this paper – to let one’s scientific endeavour start from, and be informed by, one’s own life world occurences, and to meditate upon one’s already being *in* the phenomenon (or ‘in the weave’) – could not be more striking. For Bacon, such navel-gazing has nothing to do with science. Quite the contrary: Only one who prescinds and abstracts from himself is capeable of generating scientific knowledge of the world. That way, then, the individual disappears by assuming the stance of an academic ‚we‘; his propositions appear as if issuing from a transpersonal authority, i.e., of scientific method. The objectivity of such knowledge is warranted precisely through its being independent from those who produce and communicate it (Mruck and Breuer 2003).^10^ According to this epistemology, it is only reasonable that the latter often feel responsible for neither the implementation nor the consequences of such knowledge.

In the *Novum Organum*, Bacon‘s distrust in the ‘personal side’ of knowledge is elaborated in the famous chapters on the four idols. There he delineates the reasons why human understanding is so easily led astray by the ideologies of the time, by the words that circulate in public spaces and market places; by very personal burdens or traumas that are owed to early socialization (to put this in contemporary terms); and to cognitive biases (again in contemporary terms) that are part of our evolutionary heritage. All these arguments are fundamatally convincing up to this very day. For Bacon, such daunting defects in human understanding call for strong correctives and reliable support in the form of experiments and instruments. That we should completely prescind from ourselves and that from this prescinding a methodology of a non-involved observer should emerge, of an observer who may observe the whole world except the one who is observing – this, however, is not covered by arguments put forward in the *Novum Organum*. At least one could also derive from it a plea for the necessity of a permanent epistemic reflection on the conditions of human understanding.

Intrinsically, cultural psychology does not have to be concerned too much about the issue of the ‘neutral’ observer. As longs as it understands itself as dealing with ‚acts of meaning‘(Bruner 1990), insofar as such acts make up the world of human beings, cultural psychology amounts to a primarily hermeneutic rather than an experimental endeavour. It predominantly relies on such empirical methods that give access to the realm of meaning, i.e., on qualitative methods. Contemporary qualitative researchers are mostly familiar with the idea that specifying one’s perspective (e.g. a feminist perspective) together with transparency in regard to one‘s research decisions increases the credibility of one’s findings (at least for cultured recipients). And for a hermeneutic methodology that is not totally out of sorts it is a matter of course that an interviewer cannot be subtracted from an interview, and that an interpreter cannot be subtracted from an interpretation.

This is because to understand something is to bestow meaning upon it, and meaning is endowed by (embodied) consciousness. Moreover, meaning is a relational value, i.e., the meaning of an act, a talk, an image etc. unfolds differently depending on what is used as reference or comparison point for it. Any meaning relationing begins from one’s own position – there is no other starting point for making sense. One’s own position in the cultural web of meaning, and the premises that go along with it, thus, cannot simply be bypassed. To substract the interpretor from the interpretation and to instead conceive of him as a neutral observer about whom one remains silent, does not make sense in hermeneutic methodology. Rather, understanding always comes from a perspective; it is always *my* understanding to begin with. My understanding, though, is not an erratic or accidental, private happening; it is also the understanding of a particular epoch, of *my* epoch; it is a gendered understanding, an understanding from a particular social angle, etc. The particular focusing as well as the particular blind spots that go along with such positioning are always at play in acts of understanding, starting with the questions that are asked about the particular mater (texts, images, acts) in the first place.

All this is state-of-the-art in contemporary hermeneutic methodology and not a special secret of Viennese cultural psychologists. However, if one now adds the motif that this text is circling around, some specifics of our teaching and our research topics are better understood in their structural cohesion. According to the credo of the usefulness of tracking one’s own path, in our teaching of qualitative methods we support the idea that a researchers particular perspective, as a result of her particular life situation, does not amount to a prejudiced, but rather to a distinguished observational starting point. As a result of this conviction, we call on our students, right at the beginning of their training in qualitative methods, to identify concrete irritations that occur to them in their own life worlds and to develop such irritations into research questions. This move fundamentally runs counter to the prevalent methodological training in contemporary mainstream psychology with its credo that research questions have to be delineated from (desiderata in) theory. It is a “teaching against the grain” (Eakin and Mykhalovskiy 2005). If the appropriation of qualitative methods takes place, as is mostly the case with psychology students, after thorough training in statistics and natural scientific research methodology, such an invitation – i.e., to start research from one’s own vantage point – is usually a moment where their ‘epistemological tires’ screech the loudest. When this screeching is overcome, however, and the new invitation is embraced, exceptional research energies can be mobilized which could not have been articulated before und which have just been waiting for an outlet.

In order to be able to fully accept this invitation – i.e., to develop what interests, fascinates, irritates them in the first place into a research agenda – it is crucial that students abstain from the repertoire of variables and constructs, and from the habit of looking for causal connections between such variables or constructs which represent the core of mainstream psychology’s theoretical and methodological training. Instead of being taken in by ‘false objects’ (to put this in a Foucauldian way), they have to start and stay with their own experience and with the questions that arise from this experience. And they also have to stay in touch with the language in which the social or psychological phenomena express themselves rather than bypassing such lay concepts for the sake of scientific rigor and abstractness.

We emphasize such pedagogics, as we want to pick up the description of our ‘terrain of life’ from this very terrain itself, on which we live and move and have our being. Hence, we have to start with the posing of questions that are fundamentally grounded in experience. In the realm of the social sciences and psychology, such questions are not to be geared toward what phenomena ‘are’, but rather toward how they are created, maintained, and performed. To put it in a nutshell: We invite questions of fabrication.

Contemporary mainstream psychology, by contrast, from early on rehearses the opposite (and detrimental) habit to transcend and pass over the life-worldly phenomenological givenness for a second word – a ‚world behind’ [*Hinterwelt*] in Nietzsche’s sense. This second world is all about abstracta and primary properties, which supposedly account for the phenomena; and the whole purpose of empirical research is to uncover causal or correlative relations between them. At the beginning of contemporary mainstream psychology there stands a monstrous clear-cut: a drill, in the guise of scientific necessity and rigor, to neglect the immediate phenomenal givenness and the layer of the conjunctive knowledge, to neglect the collective experiential spaces (in the sense of Mannheim 1980), in which we have always been moving and by which we have already been permeated, even in regard to our scientific ambitions and activities (Przyborski and Slunecko 2009).

Where the abstracta and the primary properties come from, on which we should restrict our observations according to contemporary mainstream psychology, is not quite transparent. In any case, they do not seem to emerge from a methodologically controlled process; usually, they are proposed in a quite freehanded manner in ‘introductory coursework’. It is by such freehand positings that most novices of contemporary psychology are quickly made familiar with psychological phenomena while, in essence, these phenomena are withdrawn from them. This is why conventional introductions to psychology or to its subfields primarily and for the most part amount to ‘deportations’. Whoever becomes acquainted with psychology’s ‘body of knowledge’ through such introductions, will rarely ever know what it could really be about (Slunecko 2012).

### The Dangers of Hasty Familiarity with False Objects and of Half-Knowledge

In the above paragraph, the word ‘familiar’ was chosen with something in mind: In his *Phenomenology of Spirit,* Hegel (1977, p. 18, § 31) asserts that “generally, the familiar, just because it is familiar, is not cognitively understood. The commonest way in which we deceive either ourselves or others about understanding is by assuming something as familiar, and accepting it on that account; […] such knowing never gets anywhere, and it knows not why”. This is so, because the way one knows something that one is only ‚familiar with‘, springs just from representation and not from saturated experience; it is not really connected with one’s existence. Contrary to such mere representation, again in Hegel’s words, “[t]he manner of study in ancient times differed from that of the modern age in that the former was the proper and complete formation of the natural consciousness. Putting itself to the test at every point of its existence, and philosophizing about everything it came across, it made itself into a universality that was active through and through. In modern times, however, the individual finds the abstract form ready-made” (Hegel 1977, p.18, §33).

This ready-made, however, because the becoming familiar with it has completely played itself out in the realm of representation, can never be a source of reassuring knowledge. No wonder that so many academic psychologists of our times, who have never learned to unfold their academic knowledge out of life-world experience, or at least connect the two in meaningful ways, are somewhat insecure in regard to the reliability of what they know. Of course, they know nothing for sure. And if they think they do, even worse! 11 “Hence the task nowadays consists not so much in purging the individual of an immediate, sensuous mode of apprehension^12^ …, but rather in just the opposite, in freeing determinate thoughts from their fixity so as to give actuality to the universal” (Hegel 1977, p.18, §33). ‘To give actuality to the universal’ – that’s easier said than done. Once a representational consciousness with ready-made content has established itself, it is difficult to get rid of it again, as „it is far harder to bring fixed thoughts into a fluid state than to do so with sensuous existence”. This is because “fixed thoughts have the ‘I’, the power of the negative, or pure actuality, for the substance and element of their existence, whereas sensuous determinations have only powerless, abstract immediacy, or being as such (Hegel 1977, p.19/20, §33).

“At the same time”, such ready-made representational consciousness is “something *familiar,* something which the existent Spirit is finished and done with, so that it is no longer active or really interested in it (Hegel 1977, *P 18 §30).* He who is already acquainted with the familiar feels no need to further question it. But can it be that the familiar, exactly because it is known only in this way – as something merely represented – and is not questioned any further, eludes us and withdraws itself? And that this withdrawal is the very core of the occidental epistemological constitution? Its pre-theoretical positing? Its morbid gain? At any rate, the suspicion that the prevalent occidental epistemology amounts to a mammoth immobilization manouevre in the service of a progressive disorder of participation – a “forgetfulness of being” (Heidegger) –, has been growing since Nietzsche and Heidegger (a concise account of this can be found in Sloterdijk 2010). „Science does not think“, as Martin Heidegger (1968, p.4) has provocatively put it; rather, at least when it comes to psychology, it obstructs the world, insofar as it fills up a space that could have been one’s own space with false, i.e. pre-fabricated and representational objects.

Such is the quality and function of the great majority of contemporary psychological doxa. More than half a century ago, Adorno critiziced such doxa, assembled from undigested set-pieces as it is, in an often-cited paragraph that is probably even more salient today: “What is half understood and half experienced is not the first stage of culture but its mortal enemy. This is so, because any elements of knowledge which are not integrated, not remelted into the continuity of consciousness, turn toxic and tend to become superstitions even if they criticize superstition as such” (Adorno 1993, p. 27). Such consciousness is divested from self-determination, a negative spirit, so to say, that has learned to disguise itself as ‘objective spirit’, however.^13^

### Implications for Teaching and Research

An inner space filled with borrowed knowledge and false objects, and lacking a proper tonus of one’s own curiosity, is not a good starting point for research, and the more so in the realm of cultural psychology. Thus cultural-psychological teaching, especially when it follows a mainstream psychological education, often carries a kind of therapeutic momentum: in the midst of their being high-jacked by half-knowledge, the point is to encourage students to let loose the grip of hastily familiarized theories and false objects, in order to re-gain access to – and trust in – their own curiosity; to encourage them to let themselves again be enmeshed with the phenomena and with the irritations that come along with them.

To keep a research diary – something that we encourage students to do from the very beginning to document and clarify the genesis of their own research questions – is fundamentally helpful in that regaining access to one’s own process as the fundament of a scientific agenda. In a more research-practical sense, such diaries allow them to document important research decisions (e.g., decisions for particular ways to access the field, for choosing a particular method of inquiry over another; or for abandoning a method in favour of another) and their own reactions in the research process. This is a tracking of one’s own path on small scale that nonetheless allows students to become familiar, from the very beginning of their cultural-psychological training, with the stance of epistemic reflexivity, of self-inquiry for ‘the sake of science’.^14^

In particular, we encourage students to start their scientific questioning with irritations that they have personally encountered, i.e., with something that has maybe shocked, disappointed, or fascinated them in their life-world. This, of course, means to break with the prevailing methodological socialization in the human and social sciences, at least in its dominant camp. Such socialization insists on the principle that research questions are to be delineated from theory, i.e., by reading journal articles and so identifying ‘research gaps’ or research desiderata. Most prospective psychologists, even if they are only in the second or third year of their studies, have already internalized a tacit epistemology according to which their own experience, their own questions, are not proper starting points of research. Such an avoidance strategy yields a considerable morbid gain: you are spared the challenge – and possible trouble – of having your own questions made pointed and reach out into social reality. 15

Such early sozialization and self-guiding of scientists has to be understood in the frame of neoliberal forms of governance. If science means to take one’s own irritations and raise or ‘harvest’ them into the realm of the general and the public, it is unlikely that such science will affirm the conditions that are causing these irritations. In that regard, mainstream psychology is part – and victim – of a neoliberal epistemological setup that precludes the possibility of formulating the interests and discomforts of one’s life-world in a way that receives the higher sanctions of science. If you don’t want to challenge the powers that be, it is always better to understand your irritations as something accidental and – even better – as deriving from your own personal ineptness, i.e., to individualize and privatize them. Just let the social sciences fabricate their irritations from within their own discourses, from their theories and results; there is no need for burdensome contact with reality, and least of all for one that runs through me as a person!

These didactics and tactics of mainstream psychology, i.e., its lack of interest in a research logic grounded in the life-world, together with its inclination toward a logic of substances (and not processes) can be plotted into a grand tableau, but one that largely eludes the consciousness of those involved. To a conspiracy theorist, all this must look like a grand scheme to immobilize a potentially ‘dangerous’ science – psychology – and transform it into an agent of the neoliberal world-order. What looks like an academic or intra-scientific battle over methods and methodology, is thus fundamentally political!

## Examples for Empirical Research under Such Methodological Premises

Within the last 10+ years, my encouragement to students to develop their irritations into empirical research projects has led to an array of diploma, master’s and PhD theses that bear witness to the extraordinary release of research energy that can result from such suggestion. Many of these projects were ignited by personal irritations, often from the student’s immediate socio-political sphere, e.g., with how the university of Vienna had been dragged into neoliberal discourse (Gartus 2008), with how easily, even in the twenty-first century, psychological knowledge about heredity can turn into racism (Preinfalk 2013), with how the German National Ethics Council talkes of intersexuality and transgender (Wanner 2015), with how women’s magazines propagate depilation (Steinicke 2013), with how the European Union runs up its homeland-security-project (Zuser 2007; Müller 2012) or conceives of the working subject in its employment policy (Wrbouschek 2007), with how neoliberal subjectifications were ‘sold’ to East Germans after the German reunification (Katzer 2014; Slunecko 2014b), with how the inaugural speech of a newly-elected president (mis)constructs the identity of one’s home country (Ruiseco 2004; Ruiseco and Slunecko 2006), or with a dissappointment over the fact that Nobel Peace Price winner Muhammad Yunus, the praised Bangladesh inventor of the micro credit system, turns out, on closer scrutiny, to be an advance agent of capital interests (Girstmair 2010).

Methodologically, these (and many other) projects are predominantly rooted in critical discourse analysis (Wodak and Krzyżanowski 2008; Reisigl 2007, 2013; Reisigl and Wodak 2016; Jäger 2009; van Leeuwen 2008; Keller 2005)^16^. More recently, this has been complemented by analysis of viscourses (Przyborski and Slunecko 2011; Wieser and Slunecko 2013; Slunecko, Ruck & Wienigk 2014; Stadlbauer 2015) and of dispositivs (Schmid 2013; see also Schmid and Slunecko 2014; Müller 2016).

There is an interesting subset of such research projects that takes the suggestion to track one’s own path even more literally and applies it to the sometimes irritating and frustrating academic training in mainstream psychology. Given the arguments put forward in earlier sections of this paper, such training – and, of course, pre-academic schooling, too – may result in ‚open bills‘which an academic final thesis is often the last chance to settle. To recapitulate the way one has been led to take in the course of one’s academic training often means to again bring to mind the semantic and epistemic ‚smog‘one had to work one’s way through in order to then be able to exhale it once and for all.

Textbooks of psychology or medicine are a much appreciated source of this manouevre.^17^ Under such premises, Schimak 2014; cf. also Slunecko 2014a) scrutinized a particularly dubious aspect of the hegemonic knowledge that had been forced upon her in her medical studies: On analysing, again within a discourse-analytical frame, what medical textbooks know of psychosomatics, it appears to her that psychosomatics has no need of enemies if it has friends like the ones that write about it in the medical textbooks she was obliged to study. In a more or less subtle way, these textbooks carry strong resentments towards etiological models outside academic medicine, more often than not in conjunction with a not so subtle misogyny.

In a similar vein, Rott (2014) analysed textbooks of clinical psychology and psychiary that were recommended to Austrian students at that time of her studies and tapped them, again in the kind of semantic slow motion that is characteristic for discourse analysis, for what they know about ‘gender identity disorders’. Above all, the authors of these textbooks know one thing for sure: who is a man and who is a woman, and that this comes as an objective fact and is not negotiable, i.e., not part of a discursive world. Parallel to Schimak’s findings, she uncovers a strong dichotomy between what these textbooks refer to as objective facts and as subjective feelings of gender, a dichotomy that easily opens discourses of incrimination against those whose subjective identity is not in line with the objective facts. This goes hand in hand with an individualization and decontextualization that is characteristic of mainstream psychology: Persons ‘concerned’ by whatever disorder – be it gender identity disorder, psychosomatic complaints, or other – are construed as being completely isolated from their social contexts, as free floating human entities. And they are construed as passive ‚sufferers’ in deplorable life-situations, with no signs of outward-oriented, active, least of all political agency. In particular, the textbook tales that clinical psychology and psychiatry offer on ‚gender identity disorders’ orient and stabilize clinical reasoning along the lines of heteronormativity. They propagate a strictly bipolar gender model and pathologize, again more or less subtle, any non-heterosexual desire. This is the way, by which – through psychological doxa – the hegemoni regulative principles of gender identity, with all their malignities, are mediated into the knowledge and discourses ef clinical experts and practicioners and, through their well-intentioned efforts, continue to bring forth the truths that they start out with.

The combined results of these two projects alone suffice to demonstrate how deeply the apparatus of clinical psychology and psychiatry is entrenched in societal truth and power games, and how thoroughly it does the discursive legwork for the powers that be. It produces a specific kind of ‚power-knowledge‘, for which the individualization and decontextualization of problems of any kind (hence the one-person-paradigm of mainstream psychology), for which the rendering of actors as passive and disempowered, for which mongering, discourses of allegation and incrimination are constitutive. This may be with particular ease demonstrated with subjects who are ‚suffering’ from gender identity disorders; the mechanisms, however, are at play throughout clinical psychology and psychiatry, and in other fields of society as well. They are the constitutive signs of the psychopolitics of our time (Han 2017).

Stadlbauer’s thesis (2015) falls into line with the above findings, and adds an important aspect. Her discourse analysis of the new generation of neuroscience-oriented textbooks of clinical psychology provides evidence for a next step in the decontextualization of psychological phenomena and disorders: Now clinical psychology is not treating human beings anymore, but neurological circuits and transmitter systems. Psychological disorders are not something to be localized in life-worlds anymore, but in the brain! And clinical psychology is all about their localization in the brain and their neuronal correlates. The subjective experience of such disorders pales beside the truth of biology – and becomes a negligible entity. The dichotomy (and hierarchization) of ‚subjective’ and ‚objective’– of feelings and facts – here reproduces itself in a new neuro-scientific guise and is widened further. A psychological disorder located in the brain and only in the brain is something per se unsocial and unpersonal, insofar as it is not at all in touch with the concerned person’s stream of consciousness and layers of experience. Rather, it is just a matter of the brain – and quite obviously, in this form it fits more easily into the course of the world.

Such decontextulization, as already reconstructed in the previous research examples, is paradigmatic for mainstream contemporary psychology, whose first and foremost premise is to isolate individuals from their social contexts (Przyborski and Slunecko 2009), from the contexts in which they originate and in which they may also regenerate. In order to accomplish such isolation, mainstream psychology has to be conceptualized correspondingly: as a science that upholds as its most self-evident premise that anything has to pass through the methodological bottleneck of the individual, and that therefore abstraction from context and disregard of situations (Slunecko 2012) are prerequisites. And as a science that is primarily interested in developing procedures and dispositifs by way of which any disturbance, any shortfall, can be ascribed to the fault of an individual and never to systemic conditions; in other words: a science by way of which world problems are to be recast as individual pathologies.

## Ramifications for ‘any future science of cultural psychology’

In this paper, I moved from the philosophical into the methodological and then into concrete research examples. In closing, I will again raise the level of abstraction in order to explicate some meta-theoretical implications of the motif that this text is circling around and to discuss some of the impulses that may follow from it for cultural psychology.

### Situated Understanding, Located Theory

To start from one’s own path and irritation, after all, signifies an – albeit theoretically legitimized – trust in one’s own perceiving and thinking, a confidence in the fact that one’s own position in the world carries an important, if not *the* important truth-function. To borrow Von Foerster and Broecker (2010) wording: we insist – and empower others to insist – on being „part of the world“, and precisely of the scientific world.

If one transponds this idea into the methodological realm, we touch on what Mannheim (1980) has described as the explicative power of socially located thinking. Along the lines of such thinking we do not want to deduce our research questions from the ‚off’ of non-located theory, because the most important accomplishment of such questions often lies in their absolute irrelevance for the course of the world. Rather, we devote our research efforts to predicaments and constellations in which we – at least some of us – actually find ourselves for real. This is the way to recapture our life-terrain, to speak with Latour (2018), a terrain that in a good part of the social and human sciences has been lost to a strangely ascetic scientific stance, a bypassing and neutralizing of the researcher. Contrary to that stance, we want to distill issues of personal relevance – not theoretical problems of unclear genesis and grounding – from the stream of our daily affairs, retrieve them in a kind of maieutic gesture, in order to make them accessible for scientific treatment.

Such a research logic, however, transcends the ‘subjective’ on principle, insofar as it knows that there is always something collective and general articulating in it. This carries important ramifications for methodology insofar as it allows to break away from the pitfall of the epistemological dichotomy ‘subjective’ versus ‘objective’ that has haunted the European history of ideas for so long (Husserl 1970). Instead, this research logic aims at reconstructing the general (one could even speak of an ‘objective’ reality if one understand this term as an indicator of intersubjective verifiability) from what at first glance seems ‘subjective’, personal, and accidental.

### More Coherence for the Field of Cultural Psychology

In my view, such considerations are particularly important for strengthening the meta-theoretical coherence of cultural psychology. Though important voices in psychology (e.g., Bruner 1990; Gergen 1994) have time and again pleaded for a non-nomothetic, culture-sensitive psychology, liberated from the Procrustean bed of hypothesis testing and from the experimental paradigm, a common denominator for the different approaches that assemble under the umbrella term ‘cultural psychology’ is not easy to find. Certainly, under this umbrella there is an overall stronger readiness to critically reflect on epistemological and methodical foundations, an affinity to qualitative methods, to the social sciences and to the humanities as compared to the mainstream of psychology. Nevertheless, the field of cultural psychology is considerably heterogeneous in terms of meta-theory and methodology (and in terms of the felt need to connect meta-theory and methodology). At this point, it can be decisive whether cultural psychology can proceed from a critique of mainstream psychology (from which it has emerged, albeit in a variety of different shades) to a more coherent programmatics.

In this context, the suggestion proposed here is to formulate the culture-psychological paradigm as the specific way of *how* this science brings forth its knowledge: namely as an access to socio-psychological worlds that is always informed by the researchers‘standpoint; through a basic trust in the knowledge potential of one’s own questions, one’s own irritability, i.e., a basic ‚yes‘to the truth value of one’s own position in being; through a re-embedding of reasoning and research into the socio-cultural context out of which we reason and do research; through a meta-theoretical understanding that does not confine generalization to a nomothetic research logic, but can have it happen stepwise, through reconstructing a more general or collective picture from what at first seems to be merely owed to accidental circumstances. A science based on such assumptions, a science that takes its impulses from our contact with the life-world, a science whose truth logic is not based in the phantasma of a correspondendence of representation (inside) and reality (outside), but is authenticated by this very contact with the life world – only such science will contribute to a „retrieving of realism” (Dreyfus and Taylor 2015) in psychological matters.

Methodological arguments akin to the ones articulated in this paper are already to be heard, in a variety of shades, among many groups of cultural-psychological researchers world-wide. For example, there is affinity of what is suggested here to a methodological proposal for (cultural) psychology recently put forward by Valsiner. As in Valsiner’s *Methodology Cycle* (Valsiner 2017, 2019), we want research questions to be born out of “phenomena known through practice” (Valsiner 2017, p .23), and especially from situations in which hitherto non-problematic social practices – or practices regarding the use of things – become irritating or fail. Stenner (2017) has recently pointed to the heuristic potential of such failures, liminal states, gaps, paradoxes, or sudden voids in social practice. The starting point proposed in this paper – irritation or failure – is somewhat more concrete and *affective* than in Valsiner’s Methodology Cycle, in which scientific questioning starts with intuition. And such irritation or failure is not – not by necessity – already educated by scientific training (as Valsiner suggests for intuition in his Methodolgy Cycle), but may well come from a surprise in one’s life-world quite apart from one’s scientific expertise. Subsequently, however, such initial irritations have to be *educated* so that feasible research questions can be spawned from them without losing the life-world phenomenon that attracted the researcher’s attention in the first place.

### Bridges to Adjacent Research Fields

Moreover, the methodological suggestions put forward in this paper can build bridges from cultural psychology to neighboring research areas. For two of them, historical and evolutionary psychology, this is quite evident, as these two research strands can be regarded as extending the timeframe of our leitmotif. Quite obviously, research in the social history of science, the social history of ideas, and in particular the social history of psychology represent aspects of a ‘tracking of paths’ that reach beyond individual life-spans and time-bound life experiences.^18^ Yet, if one extends the timeframe beyond the historical, another important task of cultural psychology comes into view: to recapitulate, but from a genuinely cultural-psychological perspective, the evolutionary path on which the human being has acquired the psychological, cognitive, and somatic properties that we find it endowed with today. Such evolutionary psychology, as the cultural psychologist sees it, must take into account the ‚mediality‘of the human being, its co-habitation or ‚dynamic constitution‘(Slunecko 2008, 2013) with the media that it has created and which unfold the sphere within which evolutionary premiums are awarded. Contrary to that, contemporary mainstream psychology and cognitive science have flattened the evolutionary perspective down to mere *natural selection,* and only think in terms of the forces of reproductive advantages that were at play in an evolutionary time window that has allegedly shut some 10.000 years ago, locking the human being since in a fixed position. Such thinking grossly underestimates the plasticity of the human being, an underestimation that is detrimetal to any understanding of the fundamental recasting of the human being that is going on today. This is because “anthropogenesis … did not take place, once and for all, in the dim and distant past, but is an ongoing occurrence, an unfinished process, in which it is decided whether the human being is becoming human, remaining human or becoming human again” (Agamben 2015, my translation^19^).

### A Therapeutic Side to Cultural Psychology?

Another possible reading of my leitmotif unfolds it into the realm of the therapeutical – of psychoanalysis, in particular. After more than a century of psychoanalysis, it is no big secret that partner or career choices are profoundly guided by unconscious forces. For the most part, however, scientists do not believe that primary processes^20^ could possibly orient their work in terms of the choice of their topics as well as of their methods, and that the good reasons they give for their choices could be pretense. Devereux (1967) has opened this perspective in principle, yet he focussed on extreme anxieties which come up during anthropological fieldwork and on the function of methods in the defense of such anxieties. But what if the manouevres of science are, as a matter of principle, co-constituted by unconscious libidinous and agressive forces? And what if by the very non-representedness of such forces in the scientific discourse the latter falls prey to ideology – falls back to myth, as Horkheimer and Adorno (1982) have warned against?

Such warning may have been spelled out every now and then, but has hardly been considered in academic programs and research institutions. To catch up with it would necessitate formats in which reflective-therapeutical elements were brought into systematic contact with scientific reasoning. It would need scientists who accept the invitation to understand their research interests in a functional relation with their biography (Thomä et al. 2015) and, perhaps, even in the light of their intrapsychic conflicts. It is by such manoeuvres – utopian as they may seem, given the present high-speed scientific production belts – that the genuine ambition of enlightenment would be reflected back upon ourselves as scientists. Otherwise much of scientific endeavor possibly remains a method to turn unreflected subjectivity – “ideologically contaminated ideas”, as Feyerabend (1975) has put it – into objective, scientifically validated truth.

It is worth noticing that our little motif here acquires a somewhat paradoxical moment: By way of reflective recapitulation we are to be brought to accept how we have become what we are and what we think, yet without forgetting that we thereby, alongside this very same track, also have become *what we are not*. I owe this nice little formula – *How I did become, what I am not* [“Wie ich wurde, was ich nicht bin”] – to an early writing by Kittler (2015) and bring it into play at the end of this treatise to make it clear that a looking for one’s tracks does not boil down to an affirmation of identity. It is not a turning back to the local, but rather transcends all identity. Insofar it also does not coincide with the classical dictum ‚Know who you are!’ – at least not in its common interpretation. Recapitulating the path that has led to what I am and what I do now rather questions my current status and practices, insofar as it exposes the twisted, the compromised, the early subjected and subordinated aspecs of my personality that underly these practices; but precisely from this very exposition new possibilities open. In a widely quoted passage, Foucault asserts that „the target nowadays is not to discover what we are, but to refuse what we are. We have to imagine and to build up what we could be … promote new forms of subjectivity through the refusal of this kind of individuality which has been imposed on us for several centuries” (Foucault 1983, p. 216).

All these are ingredients of the libido sciendi of the cultural psychologists, as I see them. As scientists who become aware of what has led to them and has led them – personally as well as historically – and from this awareness derive a new understanding of their own knowing in both its theoretical and research practical aspects, and perhaps an aesthetics of existence vis-à-vis the powers that be. Could that be the way in which Giambattista Vico’s early eighteenth-century vision of a *scienza nuova* now, after almost 300 years, finds its contemporary gestalt?

## Footnotes

1 Contrary to what one might guess from afar (given the world-wide recognition of this city as the birth-place of psychoanalysis), academic psychology in Vienna had been detached from psychoanalysis, both intellectually and institutionally, until very recently, when the Sigmund Freud Private University started a psychology program in 2007. At the University of Vienna, psychoanalysis has played virtually no role in the development of psychology – or only as its negative horizon (Benetka and Slunecko 2015). That psychoanalysis has nevertheless left its mark on the author is, therefore, not something typical for academic psychology in Vienna, but rather an exemption to an otherwise quite successful separation and suppression.

2 „[…] um von allem je Gewesenen neu Gebrauch zu machen oder erstmals das zu leben, was in ihm ungelebt blieb.“

3 „Wir stecken gleichsam in einem Urwald geschichtlicher Vorprägungen, der nicht durch bloßen Entschluß zur Unbefangenheit in ein freies Feld verwandelt werden kann“.

4 “ein bewußter Aufenthalt in der Bewegung“

5 „Wer in der Bewegtheit zu denken versucht, muss zeigen, was es bedeutet, ein Fallbeispiel zu geben.“

6 „Wer sich über das Zur-Welt-Kommen eines einzigen Individuums hinreichend radikal Rechenschaft ablegt, erreicht den Punkt, an dem die Fallgeschichte als Seinsgeschichte zu referieren ist.“

7 „Die einzige Weise, uns auf den Weg zum Universalen zu machen, ist, uns ausschließlich um das zu kümmern, was uns selber betrifft.“

8 Recapitulating or unfolding one’s personal or scientific biography (if we admit this distinction one more time) is something that can neither be done exhaustively nor completely systematical. We can never fully catch up with ourselves.

9 All this is deeply entrenched in the Christian history of ideas.

10 Bachelard has pointed to the deep inner connection between such attitude of scientific anonymity, such tacit agreement to not broach the issue of one’s own life-world experience (an agreement which has been preached to us as the highest good of science for the last 400 years), with the anonymity of a global exploitation that proceeds through (natural) scientific means.

11 For the sake of public appearance and third-party-money-acquisition, researchers are increasingly required to connect their ready-made theories and/or lab results with life-world experiences or problems. It is often on such occasions that the ideological undercurrents of such ready-made theories are most evident.

12 This can be read as a side blow to Bacon‘s distrust in immediate sensuous experience.

13 Horkheimer and Adorno (1982) saw Enlightenment not just as a source of liberation but accompanied by a shadow of oppression and unwillingness to reflect upon the conditions of its own possibility and genesis. For them, Enlightenment is, thus, destined to fall back into myth, as all totalizing worldviews do.

14 In the course of a field work under such auspices, the researcher’s previous knowledge, her prejudices as well as idealizations of the phenomena regularly undergo serious stress tests. To document them in a research diary may contribute to the therapeutic quality of the research process.

15 Our invitation for the students to anchor their research projects in their own experience, and particularly in the irritations that their social worlds provide for them, by no means implies that such research should not take hold of other knowledge resources and tools (Mruck and Breuer 2003). Basic notions, by which such research questions are articulated – e.g. style, identity, habitus, role, etc. – are to be cleared and sharpened and other metatheoretical sensitizations can be brought into play, as well, without compromising the altogether inductive/abductive orientation of the research.

16 The elective affinity of discourse analysis and cultural psychology is argued in Slunecko & Wieser (2014, p. 352f).

17 The following examples of critical textbook studies are described in much greater detail in Slunecko (2017).

18 Researchers have a tendency to look at their own local traditions first, and we also do this in Vienna (e.g., Benetka and Slunecko 2015, in press; Slunecko and Benetka 2017; Luckgei et al. in preparation). These traditions, however, are embedded in epistemic traditions of longue durée, the knowledge of which is pivotal for a genuine understanding of the forces at play.

19 „Die Anthropogenese hat sich nicht in grauer Vorzeit ein für alle Mal vollzogen; sie ist ein Ereignis, das unablässig geschieht, ein nicht abgeschlossener Prozess, in dem sich entscheidet, ob der Mensch menschlich wird oder nicht menschlich bleibt beziehungsweise wieder wird.“

20 Primary process is a notion coined by Freud to describe unconscious forces that discharge tensions and anxieties created by the clash of the pleasure and the reality principles.

## References

[CR1] Adorno T. W. (1993). Theory of Pseudo-Culture (1959). Telos.

[CR2] Agamben, G. (2015). Europa muss kollabieren [Europe has to collapse]. An interview with Giorgio Agamben. *Die Zeit*. Retrieved http://www.diezeit.de

[CR3] Bacon, F. (1620/2000). Novum organum/the new organon. Cambridge: Cambridge University Press.

[CR4] Benetka, G., & Slunecko, T. (2015). Desorientierung und Reorientierung. Zum Werden des Faches Psychologie in Wien [disorientation and reorientation. On the becoming of psychology as a scientific discipline in Vienna]. In K.A. Fröschl, G.B. Müller, T. Olechowski, & B. Schmidt-Lauber (Eds.), *Reflexive Innensichten aus der Universität. Disziplinengeschichten zwischen Wissenschaft, Gesellschaft und Politik* [reflexive insights from within the university. The history of disciplines between science, society, and politics] (pp. 267–279). Göttingen: V&R unipress, Vienna University Press.

[CR5] Benetka, G., & Slunecko, T. (in press). Ernst Machs Bedeutung für die Herausbildung einer naturwissenschaftlichen Psychologie – ein Missverständnis mit Folgen? [Ernst Mach’s importance for the shaping of psychology as a natural science – A misunderstanding with consequences]. In F. Stadler (ed.), *Ernst Mach – Leben, Werk und Wirkung* [Ernst Mach – Life, work, and impact]. Dordrecht: Springer (Vienna Circle Collection).

[CR6] Bourdieu P, Wacquant LJD (1992). An invitation to reflexive sociology.

[CR7] Bruner J (1990). Acts of meaning.

[CR8] Cioran, E. (1979). *Vom Nachteil geboren zu sein [on the disadvantage of being born]. Frankfurt*. Suhrkamp.

[CR9] Chimirri NA, Klitmøller J, Hviid P (2015). Studying the fabric of everyday life. *Outlines – Critical Practice*. Studies.

[CR10] Devereux G (1967). From anxiety to method in the behavioral sciences.

[CR11] Dreyfus, H., & Taylor, C. (2015). *Retrieving realism*. Harvard University Press.

[CR12] Eakin JM, Mykhalovskiy (2005). Teaching against the grain: The challenges of teaching qualitative research in the health sciences. Forum: Qualitative Social Research.

[CR13] Feyerabend P (1975). Against method. Outline of an anarchistic theory of knowledge.

[CR14] Foucault M, Dreyfus HL, Rabinow P (1983). The subject and power. Beyond structuralism and hermeneutics.

[CR15] Freud S (1927). Postscript to the question of lay-analysis. J. Strachey (Transl. & Ed.), *The Standard Edition of the Complete Psychological Works of Sigmund Freud,* 20.

[CR16] Gartus, A. (2008). *Das unternehmerische Selbst im Diskurs der österreichischen Universitäten* [The enterpreneurial self in the discourse of the Austrian universities]. Unpublished diploma thesis, University of Vienna.

[CR17] Gergen, K. (1994). *Realities and Relationships. Sounding in Social Construction. Cambridge, mass*. Harvard University Press.

[CR18] Girstmair, S. (2010). *The Entrepreneurial Poor“. Das Subjekt im Anti-Aid-Entwicklungsdiskurs*. Unpublished diploma thesis, University of Vienna.

[CR19] Hall, S. (1996). Introduction: Who needs ‚identity‘? In S. Hall & P. du Gay (Eds.), Questions of cultural identity (pp 1–17). London: Sage.

[CR20] Han, B.-C. (2017). *Psychopolitics: Neoliberalism and New Technologies of Power*. Brooklyn, NY: Verso.

[CR21] Hegel, G.F. (1977). *Phenomenology of spirit*. Translated by a.V. Miller. Oxford: Oxford University Press. (original work published 1806).

[CR22] Heidegger M (1968). What is called thinking?.

[CR23] Horkheimer M, Adorno TW (1982). Dialectic of enlightenment.

[CR24] Husserl, E. (1970). *The Crisis of European Sciences and Transcendental Phenomenology: An Introduction to Phenomenological Philosophy. Translated by D. Carr. Evanston: Northwestern*. University Press (original work published 1936).

[CR25] Jäger, S. (2009). *Kritische Diskursanalyse. Eine Einführung* [Critical discourse analysis. An introduction]. Duisburg: DISS.

[CR26] Katzer, S. (2014) *Die „anderen Deutschen“. Eine kritische Diskursanalyse* [The „other Germans“. A critical discourse analysis]. Berlin: LIT-Verlag.

[CR27] Keller, R. (2005). *Wissenssoziologische Diskursanalyse – Grundlegung eines Forschungsprogramms* [Discourse analysis from a sociology-of-science point of view – Foundations of a research paradigm]. Wiesbaden: VS-Verlag.

[CR28] Kittler F (2015). *Baggersee. Frühe Schriften aus dem Nachlass*. [quarry pond. Ealry writings from the estate]. Edited by T. Hron & S. Khaled.

[CR29] Latour B (2018). Down to earth: Politics in the new climatic regime.

[CR30] V. Luckgei, N. Ruck & T. Slunecko (in preparation). Feminist Psychology at the University of Vienna, 1984–2000. A Case Study of a Temporary Hub in Feminist Psychological University Teaching. The Oxford Encyclopedia of the History of Psychology.

[CR31] Mannheim K (1980). Structures of thinking.

[CR32] Mruck K, Breuer F (2003). Subjectivity and reflexivity in qualitative research. Forum Qualitative Social Research.

[CR33] Müller, D. (2016). *Citius, altius, fortius. Self-tracking als Regierungstechnologie. Eine Dispositivanalyse* [Faster, higher, stronger. Self-tracking as governmental technology]. Unpublished diploma thesis, University of Vienna.

[CR34] Müller, S. (2012). Das Migrationsmanagement der Europäischen Union – Sicherheit, Rassismus und die Konstruktion einer europäischen Identität. Kritische Diskursanalyse des europäischen Außengrenzmanagements am Beispiel der Grenzschutzagentur FRONTEX [The migration management of the European Union – Security, racism, and the construction of a European identity. A discourse analyisis of European border management using the example of the European border and coast guard agency FRONTEX]. Unpublished diploma thesis, University of Vienna.

[CR35] Preinfalk, F. (2013). *Der Diskurs zur Erblichkeit von Intelligenz. Eine Diskursanalyse zur Verschränkung von Wissenschaft und Rassismus* [The discourse of the heredity of intelligence. A discourse analysis of the entanglement of science and racism]. Unpublished diploma thesis, University of Vienna.

[CR36] Przyborski A, Slunecko T, Valsiner J, Molenaar PCM, Lyra M, Chaudhary N (2009). Against reification. Praxeological methodology and its benefits. Dynamic process methodology in the social and developmental sciences.

[CR37] Przyborski A, Slunecko T (2011). Learning to think iconically in the human and social sciences: Iconic standards of understanding as a pivotal challenge for method development. Integrative Psychological and Behavioral Science.

[CR38] Reisigl, M. (2007). *Der Wiener Ansatz der Kritischen Diskursanalyse.* [The Vienna school in critical discourse analysis]. *Forum Qualitative Sozialforschung, 8*(2). 10.17169/fqs-8.2.270.

[CR39] Reisigl M, Bayley R, Cameron R, Lucas C (2013). Critical discourse analysis. The Oxford handbook of sociolinguistics.

[CR40] Reisigl M, Wodak R, Wodak R, Meyer M (2016). The discourse-historical approach. Methods of critical discourse studies.

[CR41] Rott, V. (2014). Wahrheitsspiele der Pathologisierung im klinisch-psychologischen und psychiatrischen Diskurs über die sogenannte Geschlechtsidentitätsstörung. Eine kritische Diskursanalyse von Lehrbuchtexten [Truth games of pathologizing in the clinical-psychological and psychiatric discourse on the so-called gender identity disorder]. Unpublished diploma thesis, University of Vienna.

[CR42] Ruiseco, G. (2004). The construction of national identity in Colombia: The elites and the European heritage. A discourse-analytical approach. Unpublished diploma thesis, University of Vienna.

[CR43] Ruiseco G, Slunecko T (2006). The role of mythical European heritage in the construction of Colombian national identity. Journal of Language and Politics.

[CR44] Sartre J-P (1981). *The family idiot*. Gustave Flaubert, 1821–1857, Vol. 1.

[CR45] Schimak A (2014). *Über die Schamesröte einer jungen Turnusärztin - Die Darstellung der Psychosomatik in Lehrbüchern der Medizin* [on the blushing of a young female trainee doctor – The representation of psychosomatics in medical textbooks].

[CR46] Schmid, M. (2013). Venus Oeconomica – Liebe und Kalkül. Eine kulturpsychologische Kritik zeitgenössischer Beziehungsökonomik am Beispiel der technischen Herstellung romantischer Intimität [Venus oeconomica – Love and calculation. A cultural-psychological critique of contemporary relationship economics using the example of the technical confection of romantic intimity]. Unpublished diploma thesis, University of Vienna.

[CR47] Schmid, M., & Slunecko, T. (2014). Die gewusste Liebe. Aspekte einer wissensförmigen Herstellung romantischer Intimität [The foreknown love. Aspects of a knowledge-based confection of romantic intimity]. In K.-J. Bruder, C. Bialluch & B. Lemke (Eds.), *Machtwirkungen & Glücksversprechen. Gewalt und Rationalität in Sozialisation und Bildungsprozessen* [Effects of power and promise of happiness. Violence and rationality in socialisation and educational processes] (pp. 285–303). Gießen: Psychosozial-Verlag.

[CR48] Schmitz, H. (2007). *Der Leib, der Raum und die Gefühle* [The lived body, the space, and the feelings]. Bielefeld & Locarno: Aisthesis-Verlag.

[CR49] Schubert, K. (2018). *Freiheit als Kritik. Sozialphilosophie nach Foucault* [Freedom as critique. Social philosophy after Foucault]. Bielefeld: Transcript-Verlag.

[CR50] Sloterdijk, P. (1993). *Weltfremdheit* [Unwordlyness]. Frankfurt: Suhrkamp.

[CR51] Sloterdijk, P. (2001). Absturz und Kehre. Rede über Heideggers Denken in der Bewegung [Fall and reversement. Lecture on Heideggers thinking in motion]. In P. Sloterdijk: *Nicht gerettet. Versuche nach Heidegger* [Not saved. Essays after Heidegger] (pp. 12–81). Frankfurt: Suhrkamp.

[CR52] Sloterdijk, P. (2010). Scheintod im Denken – Von Philosophie und Wissenschaft als Übung [apparent death in thinking. Of philosophy and science as exercise]. Frankfurt: Suhrkamp.

[CR53] Sloterdijk P (2017). Not saved. Essays after Heidegger.

[CR54] Slunecko, T. (2008). *Von der Konstruktion zur dynamischen Konstitution. Beobachtungen auf der eigenen Spur* [From construction to dynamic constitution. Observations on one’s own path]. Vienna: wuv. (original work published 2002).

[CR55] Slunecko, T. (2012). Zur Kritik der Zuschauerontologie der Psychoanalyse – vorbereitende Arbeiten [Towards a critique of observer ontology in psychoanalysis – Preliminary works]. In G. Gödde & M. Buchholz (Eds.), *Der Besen, mit dem die Hexe fliegt. Wissenschaft und Therapeutik des Unbewussten* [The broom on which the whitches fly. Science and therapeutics of the unconsious], Vol. II. (pp. 563–582). Gießen: Psychosozial-Verlag.

[CR56] Slunecko, T. (2013). Der Ausbruch aus der Umwelt. Über entscheidende Momente bei der akzidentiellen Selbstherstellung von Homo sapiens [The break-out from the environment. On some decisive moments of the accidental self-fabrication of *Homo sapiens*]. In G. Jüttemann (Ed.), *Die Entwicklung der Psyche in der Geschichte der Menschheit / Auf dem Weg zu einem integrativen Ansatz* [The deveopment of the psyche in the history of mankind. Towards an integrative approach] (pp. 128–139). Lengerich: Pabst.

[CR57] Slunecko, T. (2014a). Zur Kritik der schulmedizinischen Doxa in Sachen Psychosomatik [For a critique of academic medicine’s doxa in matters of psychosomatics]. In A. Schimak: *Über die Schamesröte einer jungen Turnusärztin – Die Darstellung der Psychosomatik in Lehrbüchern der Medizin* [On the blushing of a young female trainee doctor – The representation of psychosomatics in medical textbooks] (pp. 11–13). Hamburg: Kovač.

[CR58] Slunecko, T. (2014b). Im Osten nichts Neues. Bemerkungen zu einer überfälligen Analyse der Propaganda zur deutschen Wiedervereinigung [Nothing new in the East. Remarks on an overdue anlysis of the propaganda for the German unification]. In S. Katzer: *Die „anderen Deutschen“. Eine kritische Diskursanalyse* [The “other Germans”. A critical discourse analysis] (pp 7.10). Berlin: LIT-Verlag.

[CR59] Slunecko, T. (2017). Beobachtungen auf der eigenen Spur. Bemerkungen zu einem für die Wiener kulturpsychologische Schule charakteristischen Motiv [Observations on one’s own path. Some remarks on a distinctive motif of the Viennese cultural-psychological school]. In T. Slunecko, M. Wieser & A. Przyborski (eds.), *Kulturpsychologie in Wien* [Cultural psychology in Vienna] (pp. 27–54). Vienna: facultas.

[CR60] Slunecko, T., & Benetka, G. (2017). *Zwischen Natur- und Geisteswissenschaft: Karl Bühlers Grundlegung der Psychologie* [Between natural sciences and humanities: Karl Bühlers foundation of psychology] (pp. 191–199). Dordrecht: Springer (Vienna Circle collection).

[CR61] Slunecko, T., Ruck, N., & Wienigk, B. (2014). *Panikmache. Zur bildlichen Konstruktion von Pathologie in psychologischen Lehrbüchern* [Panicmongering. The pictorial construction of pathology in psychological textbooks]. Psychologie & Gesellschaftskritik, 38, 3, 27–48.

[CR62] Slunecko T, Wieser M, Teo T (2014). Cultural psychology. Encyclopedia of critical psychology.

[CR63] Slunecko, T., Wieser, M., & Przyborski, A. (2017). *Kulturpsychologie in Wien* [cultural psychology in Vienna]. Vienna: facultas.

[CR64] Stadlbauer, K. (2015). *Hirngespinste. Über neuronale Wahrheiten psychischer Erkrankungen. Eine kritische Diskursanalyse* [Brain chimeras. A critical discourse analysis on the neuronal truths concerning mental disorders]. Unpublished diploma thesis, University of Vienna.

[CR65] Steinicke, K.M. (2013). *Die Konstruktion der "phallischen Frau" im Diskurs um die Haarentfernung in Frauenzeitschriften* [The construction of the „phallic woman” in woman‘s magazines discourse on epilation]. Unpublished diploma thesis, University of Vienna.

[CR66] Stenner P (2017). Liminality and experience. A transdisciplinary approach to the psychosocial. Basingstoke.

[CR67] Thomä D, Kaufmann V, Schmid U (2015). *Der Einfall des Lebens – Theorie als geheime Autobiografie* [the invasion of life. Theory as secret autobiography].

[CR68] Valsiner Jaan (2017). From Methodology to Methods in Human Psychology.

[CR69] Valsiner Jaan (2019). Cultural psychology as a theoretical project / La psicología cultural como proyecto teórico. Estudios de Psicología.

[CR70] Van Leeuwen T (2008). Discourse and practice. New tools for critical discourse analysis.

[CR71] Von Foerster, H., & Broecker, M. (2010). Part of the world*. Fractals of Ethics – A Drama in Three Acts. Heinz von Foerster's most extensive biography*. Self-publication. (original work published 2002).

[CR72] Wanner, L. (2015). *Hier bin ich Geschlecht, hier darf ich sein. Diskursanalyse zur Inter*-Debatte des Deutschen Ethikrates* [Here, I am gender. Here, I may be. A discours analysis of the Inter*-debate of the German Ethics Council]. Unpublished diploma thesis, University of Vienna.

[CR73] Wieser M, Slunecko T (2013). Images of the invisible: An account of iconic media in the history of psychology. Theory & Psychology.

[CR74] Wodak R, Krzyżanowski M (2008). Qualitative discourse analysis in the social sciences.

[CR75] Wrbouschek, M. (2007). *Der arbeitende Mensch im beschäftigungspolitischen Diskurs der Europäischen Union*. [The working subject in the European Union’s discourse on employment politics]. Unpublished diploma thesis, University of Vienna.

[CR76] Zuser, C.A. (2007). *Die Festung Europa wird in Afrika ausgebaut. Rassismus und die Konstruktion einer europäischen Identität*. [The fortress Europe is built in Africa. Racism and the construction of a European identity]. Unpublished diploma thesis, University of Vienna.

